# Increased both PD–L1 and PD–L2 expressions on monocytes of patients with hepatocellular carcinoma was associated with a poor prognosis

**DOI:** 10.1038/s41598-020-67497-2

**Published:** 2020-06-25

**Authors:** Hidetaka Yasuoka, Akira Asai, Hideko Ohama, Yusuke Tsuchimoto, Shinya Fukunishi, Kazuhide Higuchi

**Affiliations:** 2nd Department of Internal Medicine, Osaka Medical College, 2-7, Daigakumachi, Takatsuki, Osaka 569-8686 Japan

**Keywords:** Cancer, Tumour immunology

## Abstract

Anti-programmed cell death-1 (PD-1) antibodies has been approved to treat HCC. Some PD-1 ligands (PD–L1 and PD–L2) negative tumors respond to treatment of anti-PD-1 antibodies, and this fact may be caused by the expression of PD-1 ligands on non-tumor cells. PD–L1 was recently found to be expressed on CD14^+^ cells from cancer patients. We investigate PD-1 ligands expression on CD14^+^ cells of patients with HCC and the role of CD14^+^ cells in an antitumor response. In this study, 87 patients diagnosed with HCC were enrolled. CD14^+^ cells from patients with HCC expressed PD–L1 (4.5–95.5%) and PD–L2 (0.2–95.0%). According to cut-off values, we classified patients as those either with PD–L1^+^PD–L2^+^CD14^+^ cells or other types of CD14^+^ cells. The overall survival of patients with PD–L1^+^PD–L2^+^CD14^+^ cells was shorter than that of patients with other types of CD14^+^ cells (*p* = 0.0023). PD–L1^+^PD–L2^+^CD14^+^ cells produced IL-10 and CCL1, and showed little tumoricidal activity against HepG2 cells. The tumoricidal activity of CD8^+^ cells from patients with PD–L1^+^PD–L2^+^CD14^+^ cells were suppressed by co-cultivation with CD14^+^ cells from the syngeneic patient. Furthermore, anti-PD-1 antibody restored their tumoricidal activity of CD8^+^ cells. In conclusion, some patients with HCC have PD–L1^+^PD–L2^+^CD14^+^ cells that suppress their antitumor response. These inhibitory functions of CD14^+^ cells may be associated with a poor prognosis in these patients.

## Introduction

Hepatocellular carcinoma (HCC) is the sixth most common cancer and the fourth most common cause of cancer-related death worldwide. Most cases of HCC occur in patients with liver diseases caused by hepatitis B, hepatitis C, alcoholic hepatitis or non-alcoholic steatohepatitis (NASH)^1^. Although various treatments for HCC exist, including surgery, radiofrequency ablation, microwave coagulation therapy, percutaneous ethanol injection therapy, transcatheter arterial chemoembolization, and molecular-targeted drugs, the prognosis of patients with this disease remains poor^2–4^. In spite of patients with HCC receiving such therapies, multifocal HCC often arises synchronously, and metastasizes as new tumors or as intrahepatic metastases of the primary cancer, leading to high mortality rates^5–7^. Therefore, a new treatment for HCC is urgently needed.

Cancer cells are transformed from normal cells in response to carcinogens and other genotoxic insults in addition to the failure of intrinsic tumor suppressor mechanisms (e.g., p53, ataxia telangiectasia mutated)^8^. An immune surveillance system exists that monitors and eliminates cancer cells. However, cancer cells escape surveillance by a process known as cancer immunoediting.

Recently, a new immunosuppressive pathway, the programmed cell death 1 (PD-1) pathway that is involved in the immune evasion of cancer, has been discovered. The PD-1 pathway is activated by programmed cell death 1 ligand 1 (PD–L1) or programmed cell death 1 ligand 2 (PD–L2) binding to PD-1 expressed on T cells. Activation of the PD-1 pathway suppresses the differentiation of naïve CD8^+^ cells into cytotoxic T lymphocytes (CTLs) and also leads to the exhaustion of CTLs to finally suppress the antitumor immune response of CTLs^9–11^. The PD-1 pathway suppresses antitumor immunity by the aforementioned mechanisms. And PD–L2 expression on tumor cells may promote tumor metastasis and predict poor prognosis in solid cancer patients in HCC^12^. In recent years, reports on the expressions of PD–L1 and PD–L2 in multiple types of host cells in the tumor environment including dendritic cells, macrophages, fibroblasts and T cells have been increased^13^. There are some reports according to the correlation between the expression PD–L1 or PD–L2 in HCC and the prognosis^14–16^. However, relationship between monocytes which expressed both PD–L1 and PD–L2 and the prognosis of the diseases is unclear.

Nivolumab, an anti–PD-1 antibody, was first approved for the treatment of HCC in the U.S.A. in November 2017. The anti-PD-1 antibody augments the antitumor response of CD8^+^ cells by blocking the PD-1 pathway. Clinical trials suggest that blockade of the PD-1 pathway induces sustained tumor regression in various tumor types. More specifically, responses to anti-PD-1 antibody may correlate with the expression of PD-1 ligands by cancer cells.

However, some PD–L1-positive tumors do not respond to anti-PD-1 antibody, while a proportion of PD–L1-negative tumors react with this antibody^17,18^. Discrepancies associated with a clinical response to anti-PD-1 antibody and PD–L1 expression on the tumor are not fully understood, although several mechanisms have been suggested^19,20^.

Cancer cells that express PD–L1 suppress the antitumor response of CD8^+^ cells by activating the PD-1 pathway^11,21,22^. In recent years, monocytes/Mφ have been found to also express PD–L1^23,24^. PD–L1 is also expressed on CD14^+^ cells from patients with diverse cancers. As with CD8^+^ cells, CD14^+^ cells also kill cancer cells.

In this study, we investigated the relationship between the prognosis of HCC and the expression of PD–L1 and PD–L2 on CD14^+^ cells. We also examined the inhibitory function of monocytes/Mφ against the antitumor response of CD8^+^ cells by the PD-1 pathway, and the restorative effect of an anti-PD-1 antibody on the antitumor response of CD8^+^ cells.

## Results

### Relationship between PD–L1 and PD–L2 expression on CD14^+^ cells and patient prognoses

**Figure 1:**
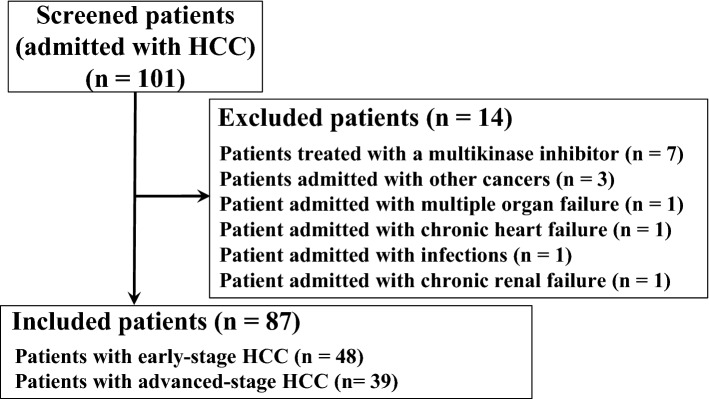
Flow chart of eligible and included patients in this study.

**Figure 2:**
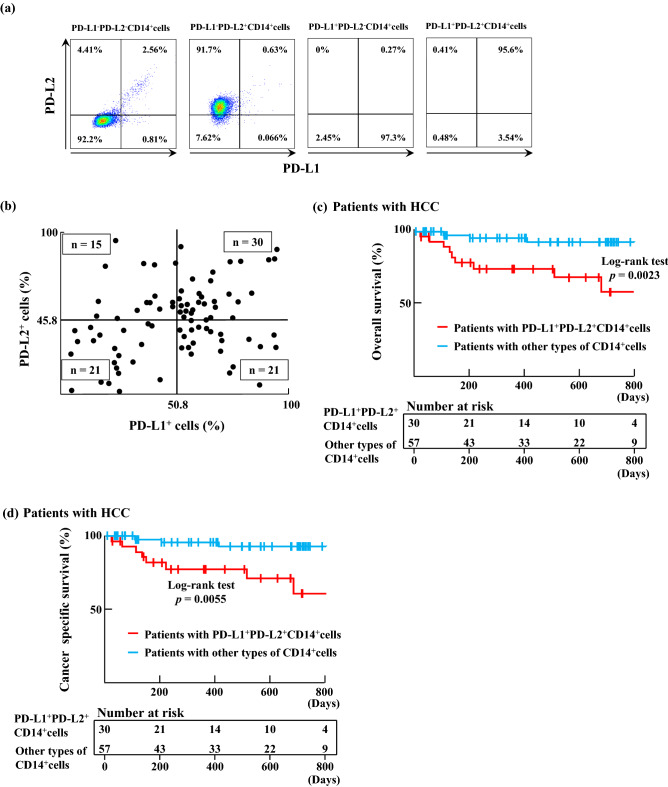
Relationship between PD–L1 and PD–L2 expression on CD14^+^ cells and patient prognoses. (**a**) CD14^+^ cells isolated from patients with hepatocellular carcinoma (HCC) were classified into four subgroups (PD–L1^+^PD–L2^+^ CD14^+^ cells, PD–L1^+^PD–L2^−^ CD14^+^ cells, PD–L1^−^PD–L2^+^ CD14^+^ cells and PD–L1^−^PD–L2^−^ CD14^+^ cells). (**b**) PD–L1 and PD–L2 expression of CD14^+^ cells from patients with HCC (n = 87). The average value of PD–L1 expression on CD14^+^ cells was 50.8%, and the average value of PD–L2 expression on CD14^+^ cells was 45.8%. (**c**) Kaplan–Meier curves for overall survival (OS) in patients with PD–L1^+^PD–L2^+^ CD14^+^ cells (n = 30; red line) and patients with other types of CD14^+^ cells (n = 57; blue line) were drawn. (**d**) Kaplan–Meier curves for cancer specific survival in patients with PD–L1^+^PD–L2^+^ CD14^+^ cells (n = 30; red line) and patients with other types of CD14^+^ cells (n = 57; blue line).

Total 101 patients were enrolled in this study and 87 patients were eligible (Fig. 1). The mean age of included patients (87 cases) was 74.1 years. Of these patients, 68 were male and 19 were female. The etiology of HCC was hepatitis B-related liver disease for 17 patients, and hepatitis C-related liver disease for 39 patients, while the HCC of 31 patients had other etiologies. Forty-eight patients presented with early-stage HCC, while the remaining 39 patients presented with advanced-stage HCC. Seventy-five, eleven and one patient had Child–Pugh class A, B, and C cirrhosis, respectively. Mean hematological values for these patients were 143.9 (ng/ml) for alpha fetoprotein (AFP), 300.3 (mAU/ml) for des-gamma-carboxyl prothrombin (DCP), 4.75 (× 10^6^ cells/ml) for the white blood cell (WBC) count, 1.28 (× 10^6^ cells/ml) for the lymphocyte count, 3.21 (× 10^4^ cells/ml) for the CD14^+^ cell count, and 4.93 (× 10^3^ cells/ml) for the CD8^+^ cell count (Table 1). CD14^+^ cells from these patients expressed PD–L1 (range 4.5–95.5%) and PD–L2 (range 0.2–95.0%; Fig. 2a,b).

**Table 1:** Clinical background of all patients.

All case	87
Age (year, range)	74.1 (46–88)
**Gender (%)**	
Male	68 (78.2)
Female	19 (21.8)
**Etiology (%)**	
HBV	17 (19.5)
HCV	39 (44.8)
Others	31(35.6)
**HCC stage (%)**	
Early	48 (55.2)
Advanced	39 (44.8)
TNM stage	
I/II/III/IV	28/25/22/12
Child–Pugh class	
A/B/C	75/11/1
AFP (ng/ml, range)	143.9 (1.0–4,266.0)
DCP (mAU/ml, range)	300.3 (9.5–9,060.2)
CRP (mg/dl, mean ± SD)	0.41 ± 0.67
WBC (× 10^6^/ml, mean ± SD)	4.75 ± 1.32
Neut (× 10^6^/ml, mean ± SD)	2.95 ± 0.96
Lymphocytes (× 10^6^/ml, mean ± SD)	1.28 ± 0.60
CD14^+^ cells (× 10^4^/ml, mean ± SD)	3.21 ± 1.18
CD8^+^ cells (× 10^4^/ml, mean ± SD)	4.93 ± 2.79
**Pretreatment**	
Surgery/RFA/TACE	22/27/15

*HBV* hepatitis B virus, *HCV* hepatitis C virus, *HCC* hepatocellular carcinoma, *PD–L1* programmed cell death 1 ligand 1, *PD–L2* programmed cell death 1 ligand 2, *AFP* alpha fetoprotein, *DCP* des-gamma-carboxyl prothrombin, *Neut* neutrophil, *CRP* C-reactive protein, *WBC* white blood cells, *SD* standard deviation, *RFA* radiofrequency ablation, *TACE* transcatheter arterial chemoembolization.

**Figure 3:**
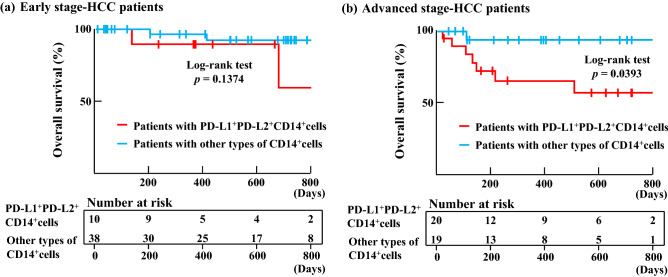
Overall survivals of HCC patients. (**a**) Kaplan–Meier curves for OS in early-stage HCC patients with PD–L1^+^PD–L2^+^ CD14^+^ cells (n = 10; red line) and early-stage HCC patients with other types of CD14^+^ cells (n = 38; blue line) were drawn. (**b**) Kaplan–Meier curves for OS in advanced-stage HCC patients with PD–L1^+^PD–L2^+^ CD14^+^ cells (n = 20; red line) and advanced-stage HCC patients with other types of CD14^+^ cells (n = 19; blue line) were drawn.

We set cut-off values as the mean value of PD–L1 (50.8%) and PD–L2 (45.8%) expression. With reference to the cut-off values, we classified patients as having either PD–L1^+^PD–L2^+^CD14^+^ cells or other types of CD14^+^ cells (PD–L1^+^PD–L2^−^CD14^+^ cells, PD–L1^-^PD–L2^+^CD14^+^ cells or PD–L1^−^PD–L2^−^CD14^+^ cells). HCC stage and TNM stage of Patients with PD–L1^+^PD–L2^+^CD14^+^ cells was different those of patients with other types of CD14^+^ cells. In this study, there was no difference in white blood cell counts and neutrophils between patients with PD–L1^+^PD–L2^+^CD14^+^ cells and other types of CD14^+^ cells (Table 3). We found that the overall survival of patients with PD–L1^+^PD–L2^+^CD14^+^ cells was significantly shorter than that of patients with other types of CD14^+^ cells (*p* = 0.0023; Fig. 2c). Cancer specific survivals (CSS) have similar results to the overall survival. CSS of patients with PD–L1^+^PD–L2^+^CD14^+^ cells was significantly shorter than that of patients with other types of CD14^+^ cells (*p* = 0.0055) (Fig. 2d). Furthermore, early-stage HCC patients with PD–L1^+^PD–L2^+^CD14^+^ cells and similar patients with other types of CD14^+^ cells did not show a significant difference in overall survival (Fig. 3a). However, overall survival of advanced-stage HCC patients with PD–L1^+^PD–L2^+^CD14^+^ cells was significantly shorter than similar patients with other types of CD14^+^ cells (*p* = 0.0393; Fig. 3b). There was no difference in the prognosis between patients with PD–L1^+^CD14^+^ cells and with PD–L1^−^CD14^+^ cells. However, compared with the prognosis of patients with PD–L1^+^PDL-2^−^CD14^+^ cells and that with PD–L1^+^PDL-2^+^CD14^+^ cells, the prognosis of patients with PD–L1^+^PD–L2^+^CD14^+^ cells was shorter than that of patients with PD–L1^+^PD–L2^−^CD14^+^ cells (SI Fig. S1). The mean age of twelve patients with HCC who died was 70.8 years. Deaths in such patients were almost cancer-related (Table 2). Of these patients, nine were male and three were female. The etiology of HCC in five cases was hepatitis B-related liver disease, for two cases it was hepatitis C-related liver disease, and for four case it was NASH-related liver disease. The mean expression of PD–L1 and PD–L2 on CD14^+^ cells from patients with HCC who died was 58.1% and 62.3%, respectively; these patients almost all had PD–L1^+^PD–L2^+^CD14^+^ cells. Thus, CD14^+^ cells from patients with HCC expressed both PD–L1 and PD–L2. Those patients expressing high levels of PD–L1 and PD–L2 on their CD14^+^ cells were found to show shorter overall survival, with most deaths being cancer-related.

**Table 2:** Clinical background of deceased patients.

No	Age	Gender	Etiology	PD–L1 (%)	PD–L2 (%)	OS (days)	Cause of death
1	65	Female	HCV	93.9	84.4	104	Cancer
2	68	Female	HCV	41	80.9	104	Cancer
3	77	Male	NASH	62.2	60.2	215	Cancer
4	75	Male	HBV	61.3	61.7	19	Cancer
5	65	Female	HBV	53.2	64.1	52	Cancer
6	72	Male	HBV	38.8	56.7	202	Cancer
7	69	Male	HBV	58.0	52.0	133	Varix rupture
8	71	Male	HBV	67.6	57.5	510	Cancer
9	77	Male	NASH	38.7	43.5	411	Cancer
10	66	Male	NASH	67.2	75.7	130	Cancer
11	67	Male	ALD	52.2	54.6	680	Cancer
12	78	Male	NASH	62.9	56.8	144	Cancer

*HBV* hepatitis B virus, *HCV* hepatitis C virus, *NASH* nonalcoholic steatohepatitis, *ALD* alcohol liver disease.

### Clinical characteristics of patients with PD–L1^+^PD–L2^+^CD14^+^ cells or other types of CD14^+^ cells

Table 3 summarizes the clinical characteristics of patients with PD–L1^+^PD–L2^+^CD14^+^ cells and patients with other types of CD14^+^ cells. The two groups of patients did not show any significant differences in age, gender, etiology, Child–Pugh class, pretreatment and hematological values (AFP, DCP, WBC count, lymphocyte count, and CD14^+^ and CD8^+^ cell counts). However, HCC stages and TNM stages in patients with PD–L1^+^PD–L2^+^CD14^+^ cells were almost all advanced, while those in patients with other types of CD14^+^ cells were mostly early stage. And, there was a significant difference in the value of CRP only. However, the value of CRP of patients with other types of CD14^+^ cells was slightly high, but the mean value of 2 or less. Interestingly, even if patients with advanced-stage cancer, patients with PD–L1^+^PD–L2^+^CD14^+^ cells also have poorer prognosis than similar patients with other types of CD14^+^ cells (Fig. 3b).

**Table 3:** Clinical characteristics of patients with PD–L1^+^PD–L2^+^ CD14^+^ cells and those with other types of CD14^+^ cells.

	Patients with PD–L1^+^PD–L2^+^ CD14^+^ cells	Patients with other types of CD14^+^ cells	*p* value
All cases	30	57	
Age (year, range)	75 (63–87)	73.7 (46–88)	0.7374
**Gender (%)**			0.4289
Male	22 (73.3)	46 (80.7)	
Female	8 (26.7)	11 (19.3)	
**Etiology (%)**			0.5397
HBV	6 (20.0)	11 (19.3)	
HCV	11 (36.7)	28 (49.1)	
Others	13 (43.3)	18 (31.6)	
**HCC stage (%)**			0.0036
Early	10 (33.3)	38 (66.7)	
Advanced	20 (66.7)	19 (33.3)	
**TNM stage**			0.0024
I/II/III/IV	3/13/7/7	25/12/15/5	
**Child–Pugh class**			0.2896
A/B/C	24/5/1	51/6/0	
AFP (ng/ml, range)	207.6 (1.5–2,969.0)	113.2 (1–4,266.0)	0.1624
DCP (mAU/ml, range)	169.9 (15.4–865.7)	359.7 (9.5–9,060.2)	0.4455
CRP (mg/dl, mean ± SD)	0.61 ± 0.81	0.31 ± 0.56	0.0055
WBC (× 10^6^/ml, mean ± SD)	4.52 ± 1.49	4.88 ± 1.22	0.2512
Neut (× 10^6^/ml, mean ± SD)	2.87 ± 0.95	2.99 ± 0.97	0.7109
Lymphocytes (× 10^6^/ml, mean ± SD)	1.19 ± 0.63	1.32 ± 0.59	0.3416
CD14^+^ cells (× 10^4^/ml, mean ± SD)	3.09 ± 1.05	3.28 ± 1.24	0.5890
CD8^+^ cells (× 10^4^/ml, mean ± SD)	4.08 ± 1.59	5.59 ± 3.34	0.3447
**Pretreatment**			0.3418
Surgery/RFA/TACE	8/8/8	14/19/7	

*AFP* alpha fetoprotein, *DCP* des-gamma-carboxyl prothrombin, *Neut* neutrophil, *CRP* C-reactive protein, *WBC* white blood cells, *SD* standard deviation, *RFA* radiofrequency ablation, *TACE* transcatheter arterial chemoembolization.

### CD14^+^ cell properties of PD–L1^+^PD–L2^+^CD14^+^ cells and other types of CD14^+^ cells

**Figure 4:**
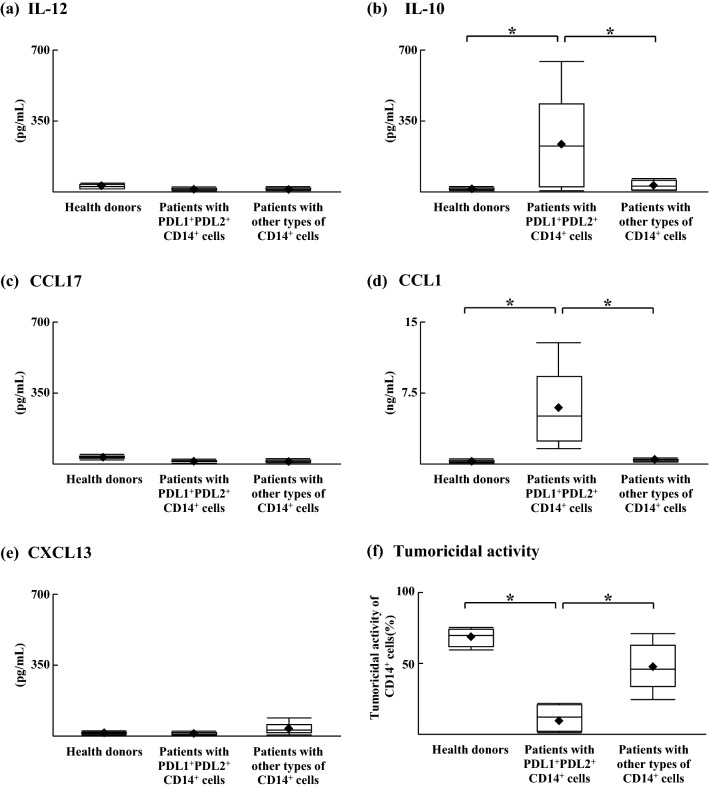
CD14^+^ cell properties of HCC patients. PD–L1^+^PD–L2^+^ CD14^+^ cells or other types of CD14^+^ cells were cultured for 24 h. Culture fluids obtained were assayed for (**a**) IL-12, (**b**) IL-10, (**c**) CCL17, (**d**) CCL1, and (**e**) CXCL13 by ELISA. (**f**) The tumoricidal activities of CD14^+^ cells against HepG2 cells were calculated by a lactate dehydrogenase (LDH) release assay. *p < 0.05.

The mean IL-12 level in the culture fluid of other types of CD14^+^ cells was as same as that of PD–L1^+^PD–L2^+^CD14^+^ cells (Fig. 4a). In contrast, the mean IL-10 level in the culture fluid of other types of CD14^+^ cells was lower than that of PD–L1^+^PD–L2^+^CD14^+^ cells (Fig. 4b). Both groups of CD14^+^ cells did not produce CCL17 and CXCL13, while only PD–L1^+^PD–L2^+^CD14^+^ cells produced CCL1 (Fig. 4c–e). In SI Fig. S2, the cytokine secretion and tumoricidal activity of each group of CD14^+^ cells (PD–L1^+^PD–L2^+^CD14^+^ cells, PD–L1^+^PD–L2^−^CD14^+^ cells, PD–L1^−^PD–L2^+^CD14^+^ cells and PD–L1^−^PD–L2^−^CD14^+^ cells) were described. Although, other types of CD14^+^ cells showed tumoricidal activities against HepG2 cells (48.0 ± 15.4%), PD–L1^+^PD–L2^+^CD14^+^ cells showed very little tumoricidal activities against HepG2 cells (10.8 ± 9.8%; Fig. 4f). Similarly, the tumoricidal activity of PD–L1^+^PD–L2^+^CD14^+^ cells against Huh7 cells was lower than that of other types of CD14^+^ cells (SI Fig. S3). These results indicated that PD–L1^+^PD–L2^+^CD14^+^ cells expressed M2b phenotypic properties and these cells did not have the tumoricidal activity against hepatocellular carcinoma.

### Anti-PD-1 antibody restored tumoricidal activities of CD8^+^ cells suppressed by PD–L1^+^PD–L2^+^CD14^+^ cells isolated from the same patient

**Figure 5:**
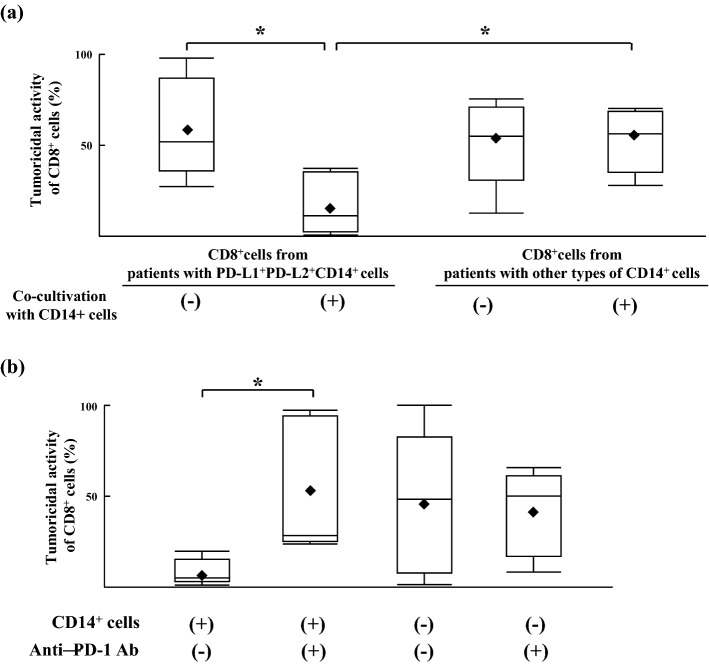
Anti–PD-1 antibody restored tumoricidal activities of CD8^+^ cells suppressed by PD–L1^+^PD–L2^+^CD14^+^ cells from the same patient. (**a**) CD8^+^ cells and CD14^+^ cells were isolated from the same patients with PD–L1^+^PD–L2^+^ CD14^+^ cells (n = 5). CD8^+^ cells (5 × 10^5^ cells/ml) and CD14^+^ cells (5 × 10^5^ cells/ml) were separately stimulated by HepG2 homogenates (corresponding to 2 × 10^5^ cells/ml) for 24 h. After washing, CD8^+^ cells were co-cultured with CD14^+^ cells or without. 24 h after co-cultivation, CD8^+^ cells were isolated and the tumoricidal activities of these CD8^+^ cells against HepG2 cells were measured by LDH release assay. And, tumoricidal activity of CD8^+^ cells from patients with other types of CD14^+^ cells (n = 5) were measured by the same methods. (**b**) CD8^+^ cells (n = 5) and CD14^+^ cells from the same patients were separately stimulated by HepG2 homogenates for 24 h. After washing, CD8^+^ cells that were co-cultured with CD14^+^ cells under the stimulation of anti-PD-1 antibody (50 μg/ml) or without for 24 h. After washing CD8^+^ cells again, the tumoricidal activities of these CD8^+^ cells against HepG2 cells calculated by the LDH release assay.*p < 0.05 (Mann–Whitney *U* test).

CD8^+^ cells isolated from patients with PD–L1^+^PD–L2^+^CD14^+^ cells showed tumoricidal activities against HepG2 cells (59.0 ± 25.0%). After CD8^+^ cells were co-cultured with PD–L1^+^PD–L2^+^CD14^+^ cells isolated from the syngeneic patient for 24 h, their tumoricidal activities were strongly suppressed (16.3 ± 16.0%). However, CD8^+^ cells isolated from patients with other types of CD14^+^ cells showed the same levels of tumoricidal activities against HepG2 cells, with or without co-cultivation with other types of CD14^+^ cells isolated from the syngeneic patients (Fig. 5a). In SI Fig. S4a, the tumoricidal activity of CD8^+^cells from each group of patients with other types of CD14^+^ cells (patients with PD–L1^+^PD–L2^−^CD14^+^ cells, patients with PD–L1^−^PD–L2^+^CD14^+^ cells or patients with PD–L1^−^PD–L2^−^CD14^+^ cells) were described. These results suggested that PD–L1^+^PD–L2^+^CD14^+^ cells suppressed the tumoricidal activities of CD8^+^ cells against HepG2 cells isolated from the syngeneic patient.

Next, CD8^+^ cells isolated from patients with PD–L1^+^PD–L2^+^CD14^+^ cells were co-cultured with PD–L1^+^PD–L2^+^CD14^+^ cells from the syngeneic patients under the stimulation of anti-PD-1 antibody. Anti-PD-1 antibody restored the tumoricidal activities of CD8^+^ cells co-cultured with PD–L1^+^PD–L2^+^CD14^+^ cells (53.3 ± 33.7%) to the same levels as those of CD8^+^ cells that were cultured without PD–L1^+^PD–L2^+^CD14^+^ cells (Fig. 5b). In supplemental Fig. 4b, tumoricidal activity of CD8^+^ cells from each group of patients with other types of CD14^+^ cells (patients with PD–L1^+^PD–L2^−^CD14^+^ cells, patients with PD–L1^−^PD–L2^+^CD14^+^ cells or patients with PD–L1^−^PD–L2^−^CD14^+^ cells) did not change, whether these CD8^+^ cells were stimulated with anti-PD-1 antibody. Thus, PD–L1^+^PD–L2^+^CD14^+^ cells suppressed the tumoricidal activities of syngeneic CD8^+^ cells against HepG2 cells in a PD-1-dependent manner.

## Discussion

Recently, the prognosis of patients with CD14^+^ cells expressing a high level of PD–L1 was found to be poor in patients with ovarian or cervical cancers^23,24^. However, few studies exist on PD–L2 expression by CD14^+^ cells from patients with diverse cancers. In our study, the prognosis of HCC patients with PD–L1^+^PD–L2^+^CD14^+^ cells was poorer than that of patients with other types of CD14^+^ cells. To the best of our knowledge, this is the first report showing that PD–L2 expression on CD14^+^ cells was associated with a poor prognosis in patients with HCC. Of these patients, we compared the prognosis of advanced-stage HCC patients with PD–L1^+^PD–L2^+^CD14^+^ cells to that of such patients with other types of CD14^+^ cells. The prognosis of advanced-stage HCC patients with PD–L1^+^PD–L2^+^CD14^+^ cells was poorer than that of similar patients with other types of CD14^+^ cells. Thus, this suggests that PD–L1^+^PD–L2^+^CD14^+^ cells may be a predictive marker of a poor prognosis in HCC patients. However, compared to other studies on the prognosis of patients with diverse cancers, this study had a relatively short follow-up period; a longer follow-up is required in future.

Classically activated Mφ (M1Mφ), which are IL-12-producing and IL-10-non-producing Mφ, are major effector cells in innate immune responses^25,26^. In contrast, alternatively activated Mφ (M2Mφ) produce IL-10 but not IL-12 and have a reduced capacity to kill tumor cells^27,28^. These latter cells suppressed the tumoricidal activities of other type 1 tumor–killing cells (M1Mφ, CTLs, NK cells and DCs)^29^. Three different subtypes of M2Mφ (M2aMφ, M2bMφ and M2cMφ) have been described and can be distinguished from each other by chemokine profile^30,31^. M2bMφ produce IL-10 and CCL1 and are thought to predominate in patients with advanced-stage HCC^7^. In this study, PD–L1^+^PD–L2^+^CD14^+^ cells produced IL-10 and CCL1, but did not have tumoricidal activities. Therefore, these cells expressed an M2b-like phenotype; the PD-1 pathway may be one of mechanisms by which M2bMφ suppresses tumoricidal activities of other type 1 tumor-killing cells.

PD–L1^+^PD–L2^+^CD14^+^ cells suppressed the tumoricidal activities of CD8^+^ cells from the same individual; anti-PD-1 antibody restored tumoricidal activities. However, paradoxically, PD–L1 negative tumors have been observed to sometimes respond to anti-PD-1 antibody^17,18^. Three primary reasons may exist for why this has been observed: The first may be the presence of a false negative caused by technical issues (e.g., inadequate sampling of the tumor, or insufficient sensitivity of the detection technique that is used). A second reason may be immune cell inhibition through PD–L2. A third reason may be the existence of a potential role for the PD-1 pathway outside of the tumor microenvironment^19,32,33^. In the present study, we found a mechanism whereby coincubation of CD8^+^ cells with PD–L1^+^PD–L2^+^CD14^+^ cells led to the inhibition of tumoricidal activities by the latter; an anti-PD-1 antibody restored tumoricidal activities. The inhibition of tumoricidal activities may be associated with the binding of PD–L1 or PD–L2 on CD14^+^ cells to PD-1 on CD8^+^ cells, while the restorative effect of anti-PD-1 antibody may be associated with competitive binding of the antibody with PD–L1 or PD–L2 on CD14^+^ cells. This suggests a mechanism by which PD–L1-negative tumors may show an apparent response to anti-PD-1 antibody. However, in vitro results only form the basis of this study. Further studies are required to measure PD–L1 and PD–L2 expression on CD14^+^ cells in patients with HCC prior to treatment with anti-PD-1 antibody and to confirm the effectiveness of anti-PD-1 antibody on such patients.

In our study, we used tumoricidal activity against HepG2 cell line. This cell line is derived from hepatoblastoma and not from hepatocellular carcinoma. There are many liver cancer lines and Huh7 cell line is one of the most famous cell lines from hepatocellular carcinoma. It is reported that there was a different response against chemotherapy and hypoxia between HepG2 cell line and Huh7 cell line^34^. However, PD–L1 expressions of cancer cell lines were not different in HepG2 cell line and Huh7 cell line^35^. Therefore, there may be no difference between those cell lines regarding the immune response through PD-1 pathway targeted in this study. The further experiments are needed in this point.

In summary, PD–L1^+^PD–L2^+^CD14^+^ cells may suppress the antitumor response of some patients with HCC. Such inhibitory functions of CD14^+^ cells may be associated with a poor prognosis in such patients. However, studies on bigger patient cohorts are required to draw more definitive conclusions.

## Materials and methods

### Ethics statement

The study was approved by the Institutional Review Board of the Osaka Medical College (IRB approved number: 2127). Written informed consent for this research was obtained from all patients. All experiments were conducted in accordance with the relevant guidelines and regulations of Osaka Medical College.

### Patients and specimens

One hundred and one patients with HCC who were hospitalized at Osaka Medical College Hospital from October 2017 to June 2019 were enrolled in this study. In this study, all patients have never been treated or have received only one treatment. Patients were classified into either an early-stage group, which consisted of patients diagnosed as very early-stage or early-stage HCC according to the Barcelona Clinic Liver Cancer (BCLC) staging system, or an advanced-stage group, which consisted of patients diagnosed as intermediate-stage or advanced-stage HCC. The following 14 patients were excluded: patients with primary or secondary immunodeficiencies (e.g., other cancers, autoimmune diseases, hematologic diseases, infections, chronic heart failure, chronic renal failure, multiple organ failure); and those who were receiving multikinase inhibitors or immunosuppressive agents (Fig. 1).

### Materials

Anti-CD14 magnetic particles-DM, anti-CD8 magnetic particles-DM and IMag buffer were purchased from BD Biosciences (San Jose, CA, USA). Phycoerythrin (PE)-conjugated anti-human PD–L1 monoclonal antibody (mAb), allophycocyanin (APC)-conjugated anti-human PD–L2 mAb, interleukin(IL)-12 ELISA MAX kits and IL-10 ELISA MAX kits were purchased from BioLegend (San Diego, CA, USA). Human recombinant C–C motif chemokine ligand 1 (rCCL1), rCCL17, and recombinant C–X–C motif chemokine 13 (rCXCL13) were purchased from PeproTech (Rocky Hill, NJ, USA). Anti-CCL17 mAbs, anti-CCL1 mAbs, and anti-CXCL13 mAbs were purchased from R&D Systems (Minneapolis, MN, USA). Cytotoxicity detection kits (lactate dehydrogenase [LDH] release assay) were purchased from Roche Diagnostics (Mannheim, Germany). We performed all experiments using these kits according to the protocol of the manufacturer. Anti-PD-1 (pembrolizumab) humanized antibody was purchased from BioVision (Milpitas, CA, USA). HepG2 cells (human hepatocellular carcinoma cells), purchased from DS Pharma Biomedical (Osaka, Japan), were cultured at 37 °C in HepG2 human hepatocellular carcinoma expansion medium (Cellular Engineering Technologies Inc., Coralville, IA, USA). RPMI-1640 medium supplemented with 10% fetal bovine serum was used for CD14^+^ and CD8^+^ cells.

### Isolation of CD14^+^ and CD8^+^ cells

Blood samples were obtained at the time of admission for the operation. Ten ml whole blood was drawn into a vacutainer tube containing a small amount of sodium heparin at the same time as a patient examination was conducted at admission. Peripheral blood mononuclear cells (PBMC) were isolated from heparinized whole blood by Lymphocyte Separation Medium 1,077 density gradient centrifugation. PBMC (5 × 10^6^ cells/ml) in IMag buffer were incubated with magnetic beads coated with anti-CD14 mAb (40 min at 4 °C) and CD14^+^cells subsequently magnetically harvested. When these CD14^+^ cells were stained for CD16 and CD68, most of these cells were CD14^+^CD16^−^CD68^−^ cells (SI Fig. S5). CD8^+^ cells were magnetically harvested using a similar method.

### CD14^+^ cell characterization

CD14^+^ cells in FACS buffer were incubated with PE-conjugated anti-human PD–L1, APC-conjugated anti-human PD–L2, or isotype control mAb for 15 min at 4 °C. After washing, PD–L1 and PD–L2 expression of CD14^+^ cells were measured by FACSAria flow cytometer and analyzed by FlowJo software ver. 10.6.0. In some experiments, CD14^+^ cells (1 × 10^6^ cells/ml) were cultured for 24 h. Culture fluids obtained were assayed for IL-12 (M1Mφ biomarker), IL-10 (M2Mφ biomarker), CCL17 (M2aMφ biomarker), CCL1 (M2bMφ biomarker) and CXCL13 (M2cMφ biomarker) by ELISA.

Next, CD14^+^ cells (5 × 10^5^ cells/ml) were stimulated by HepG2 homogenates for 24 h. HepG2 homogenates were made by crushing HepG2 cells (2 × 10^6^ cells/ml) in PBS using an ultrasonic crusher for 15 min. After washing, CD14^+^ cells were co-cultured with HepG2 cells (1 × 10^5^ cells/ml) for 24 h. The tumoricidal activities of CD14^+^ cells against HepG2 cells were calculated by LDH release assay.

### Co-cultivation and tumoricidal activities of CD8^+^ cells

CD8^+^ cells (5 × 10^5^ cells/ml) and CD14^+^ cells (5 × 10^5^ cells/ml) from the same patients were separately stimulated by HepG2 homogenates (corresponding to 2 × 10^5^ cells/ml) for 24 h. After washing, CD8^+^ cells co-cultured with CD14^+^ cells under no stimulation for 24 h. After washing again, CD8^+^ cells obtained (5 × 10^5^ cells/ml) and HepG2 cells (1 × 10^5^ cells/ml) were mixed in the U-bottomed wells of a 96-well microplate for 24 h and the tumoricidal activities of CD8^+^ cells against HepG2 cells calculated by the LDH release assay. Tumoricidal activity of CD8^+^ cells against Huh7 cells were assayed for same methods. For the experiments for anti-PD-1 antibody, CD8^+^ cells (5 × 10^5^ cells/ml) and CD14^+^ cells (5 × 10^5^ cells/ml) were separately stimulated by HepG2 homogenates (corresponding to 2 × 10^5^ cells/ml) for 24 h. After washing, CD8^+^ cells that were co-cultured with CD14^+^ cells (5 × 10^5^ cells/ml) under the stimulation of anti–PD-1 antibody (50 μg/ml) for 24 h. After washing again, CD8^+^ cells of each group were obtained. These CD8^+^ cells (5 × 10^5^ cells/ml) and HepG2 cells (1 × 10^5^ cells/ml) were mixed in the U-bottomed wells of a 96-well microplate for 24 h and the tumoricidal activities of CD8^+^ cells against HepG2 cells calculated by the LDH release assay.

### Statistical analyses

Statistical analyses were performed using JMP Pro software ver. 14 (Tokyo, Japan).

Quantitative values are expressed as means. Differences in quantitative values between two groups were analyzed using a Mann–Whitney *U* test. Differences in ratios between two groups were analyzed using Fisher’s exact test. For survival analysis, the Kaplan–Meier method was used to analyze overall survival and a log-rank test was used for comparisons. With box charts, the horizontal lines denoted median values and closed diamonds denoted the mean values. Results were considered significant if the *p* value was < 0.05.

## Supplementary information

Supplementary file1

Supplementary file2

Supplementary file3

Supplementary file4

Supplementary file5
