# Supplementary material for: Increased both PD–L1 and PD–L2 expressions on monocytes of patients with hepatocellular carcinoma was associated with a poor prognosis
Source: Sci Rep. 2020 Jun 25;10:10377. doi: 10.1038/s41598-020-67497-2 (PMC7316832; doi:10.1038/s41598-020-67497-2)
Supplement: Supplementary file 1 — Supplementary file1 [file 41598_2020_67497_MOESM1_ESM.pptx]

## Slide 1
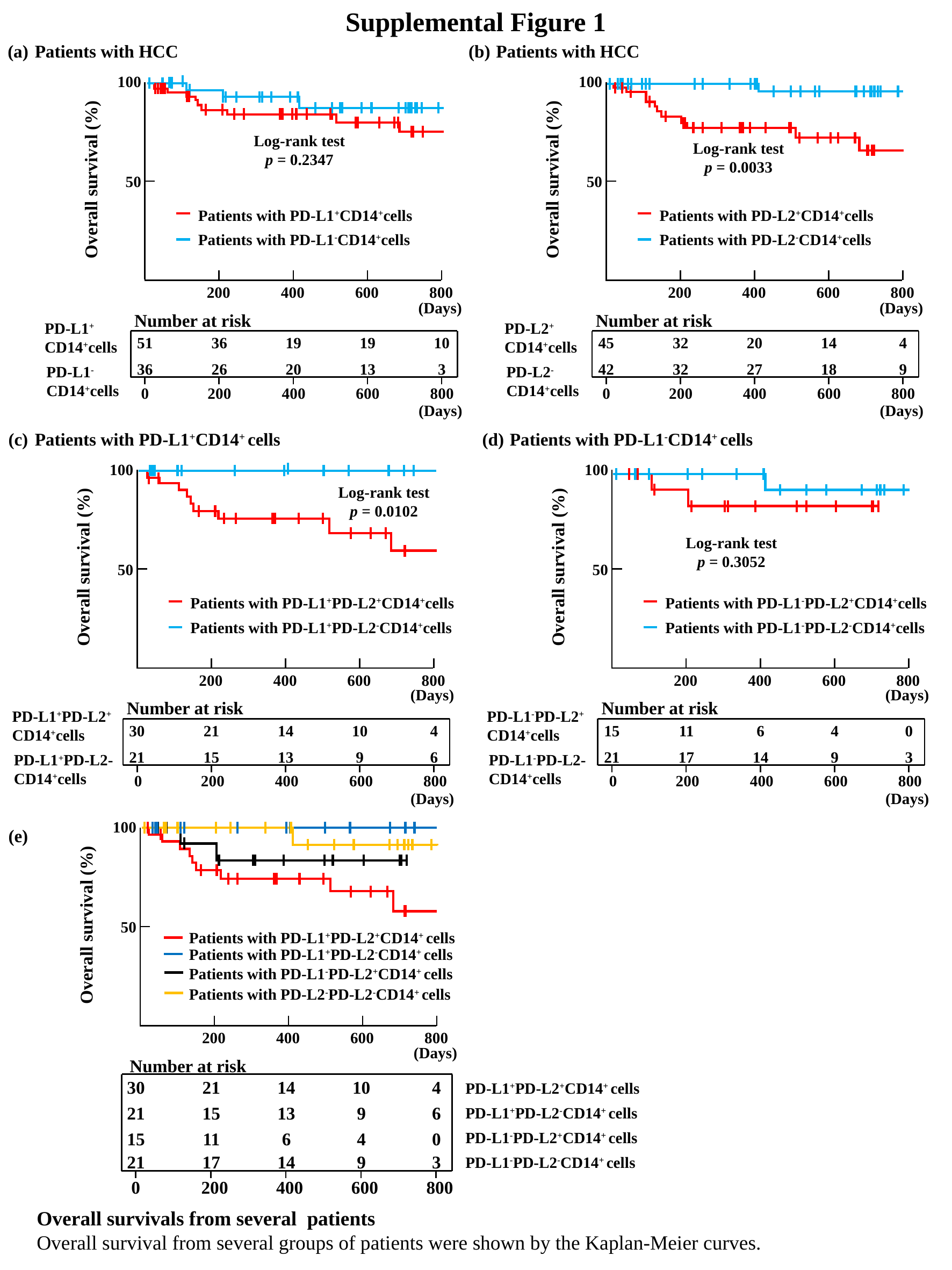

Supplemental Figure 1
(a)
Patients with HCC
100
Overall survival (%)
Log-rank test
p = 0.2347
50
Patients with PD-L1+CD14+cells
Patients with PD-L1-CD14+cells
200
400
600
800
(Days)
Number at risk
PD-L1+
CD14+cells
51
36
19
19
10
36
26
20
13
3
PD-L1-
CD14+cells
0
200
400
600
800
(Days)
(b)
Patients with HCC
100
Overall survival (%)
Log-rank test
p = 0.0033
50
Patients with PD-L2+CD14+cells
Patients with PD-L2-CD14+cells
200
400
600
800
(Days)
Number at risk
PD-L2+
CD14+cells
45
32
20
14
4
42
32
27
18
9
PD-L2-
CD14+cells
0
200
400
600
800
(Days)
(c)
Patients with PD-L1+CD14+ cells
100
Overall survival (%)
Log-rank test
p = 0.0102
50
Patients with PD-L1+PD-L2+CD14+cells
Patients with PD-L1+PD-L2-CD14+cells
200
400
600
800
(Days)
Number at risk
PD-L1+PD-L2+
CD14+cells
30
21
14
10
4
21
15
13
9
6
PD-L1+PD-L2-
CD14+cells
0
200
400
600
800
(Days)
(d)
Patients with PD-L1-CD14+ cells
100
Overall survival (%)
Log-rank test
p = 0.3052
50
Patients with PD-L1-PD-L2+CD14+cells
Patients with PD-L1-PD-L2-CD14+cells
200
400
600
800
(Days)
Number at risk
PD-L1-PD-L2+
CD14+cells
15
11
6
4
0
21
17
14
9
3
PD-L1-PD-L2-
CD14+cells
0
200
400
600
800
(Days)
100
(e)
Overall survival (%)
50
Patients with PD-L1+PD-L2+CD14+ cells
Patients with PD-L1+PD-L2-CD14+ cells
Patients with PD-L1-PD-L2+CD14+ cells
Patients with PD-L2-PD-L2-CD14+ cells
200
400
600
800
(Days)
Number at risk
30
21
14
10
4
PD-L1+PD-L2+CD14+ cells
21
15
13
9
6
PD-L1+PD-L2-CD14+ cells
PD-L1-PD-L2+CD14+ cells
15
11
6
4
0
21
17
14
9
3
PD-L1-PD-L2-CD14+ cells
0
200
400
600
800
Overall survivals from several patients
Overall survival from several groups of patients were shown by the Kaplan-Meier curves.
