# Supplementary material for: Increased both PD–L1 and PD–L2 expressions on monocytes of patients with hepatocellular carcinoma was associated with a poor prognosis
Source: Sci Rep. 2020 Jun 25;10:10377. doi: 10.1038/s41598-020-67497-2 (PMC7316832; doi:10.1038/s41598-020-67497-2)
Supplement: Supplementary file 2 — Supplementary file2 [file 41598_2020_67497_MOESM2_ESM.pptx]

## Slide 1
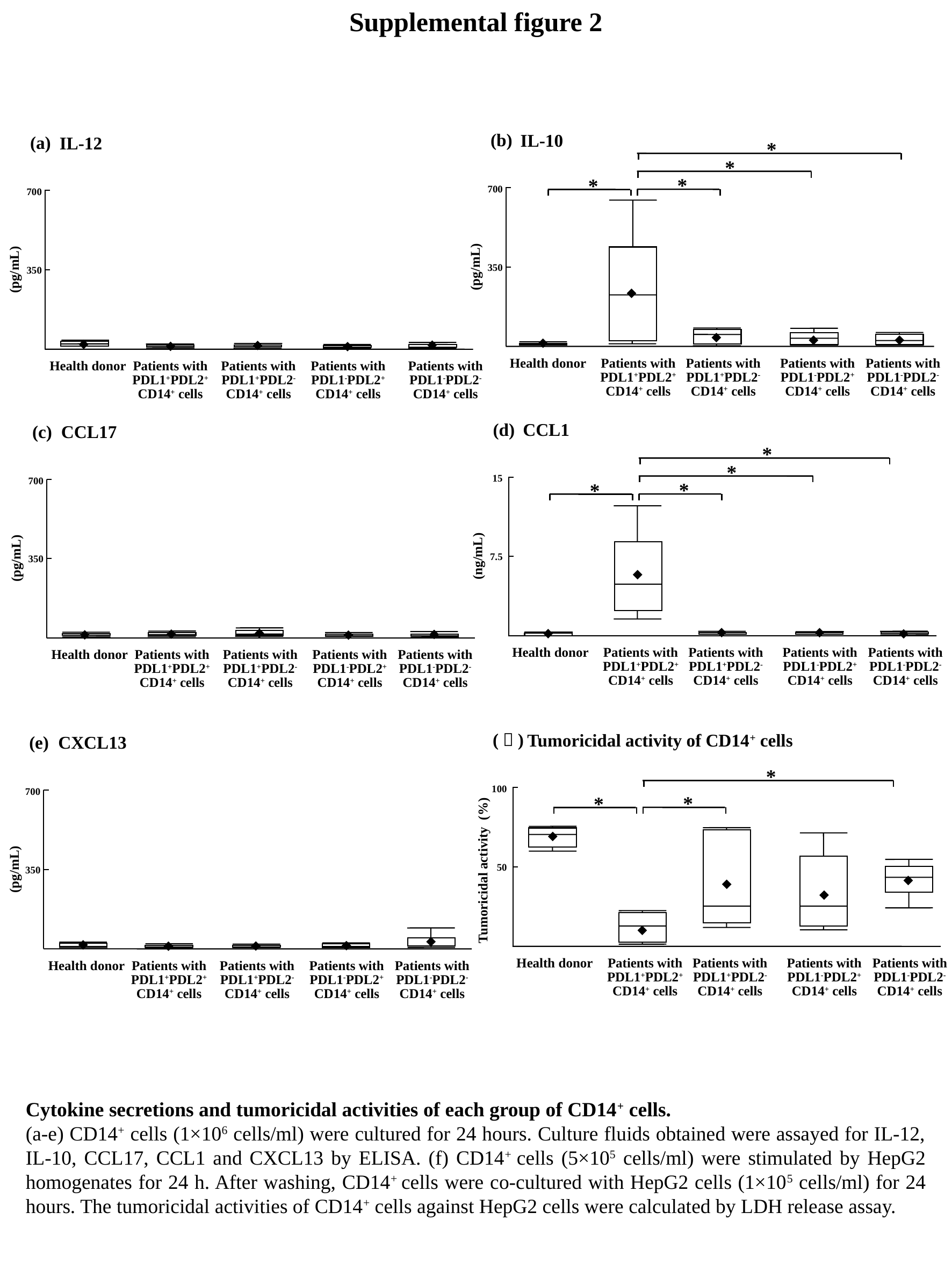

Supplemental figure 2
(b)
IL-10
(a)
IL-12
*
*
*
*
700
700
(pg/mL)
350
(pg/mL)
350
Health donor
Patients with
PDL1+PDL2+
CD14+ cells
Patients with
PDL1+PDL2-
CD14+ cells
Patients with
PDL1-PDL2+
CD14+ cells
Patients with
PDL1-PDL2-
CD14+ cells
Health donor
Patients with
PDL1+PDL2+
CD14+ cells
Patients with
PDL1+PDL2-
CD14+ cells
Patients with
PDL1-PDL2+
CD14+ cells
Patients with
PDL1-PDL2-
CD14+ cells
(d)
CCL1
(c)
CCL17
*
*
15
700
*
*
(ng/mL)
(pg/mL)
7.5
350
Health donor
Patients with
PDL1+PDL2+
CD14+ cells
Patients with
PDL1+PDL2-
CD14+ cells
Patients with
PDL1-PDL2+
CD14+ cells
Patients with
PDL1-PDL2-
CD14+ cells
Health donor
Patients with
PDL1+PDL2+
CD14+ cells
Patients with
PDL1+PDL2-
CD14+ cells
Patients with
PDL1-PDL2+
CD14+ cells
Patients with
PDL1-PDL2-
CD14+ cells
(ｆ)
Tumoricidal activity of CD14+ cells
(e)
CXCL13
*
100
700
*
*
50
(pg/mL)
Tumoricidal activity (%)
350
Health donor
Patients with
PDL1+PDL2+
CD14+ cells
Patients with
PDL1+PDL2-
CD14+ cells
Patients with
PDL1-PDL2+
CD14+ cells
Patients with
PDL1-PDL2-
CD14+ cells
Health donor
Patients with
PDL1+PDL2+
CD14+ cells
Patients with
PDL1+PDL2-
CD14+ cells
Patients with
PDL1-PDL2+
CD14+ cells
Patients with
PDL1-PDL2-
CD14+ cells
Cytokine secretions and tumoricidal activities of each group of CD14+ cells.
(a-e) CD14+ cells (1×10^6^ cells/ml) were cultured for 24 hours. Culture fluids obtained were assayed for IL-12, IL-10, CCL17, CCL1 and CXCL13 by ELISA. (f) CD14+ cells (5×10^5^ cells/ml) were stimulated by HepG2 homogenates for 24 h. After washing, CD14+ cells were co-cultured with HepG2 cells (1×10^5^ cells/ml) for 24 hours. The tumoricidal activities of CD14+ cells against HepG2 cells were calculated by LDH release assay.
