# Supplementary material for: Increased both PD–L1 and PD–L2 expressions on monocytes of patients with hepatocellular carcinoma was associated with a poor prognosis
Source: Sci Rep. 2020 Jun 25;10:10377. doi: 10.1038/s41598-020-67497-2 (PMC7316832; doi:10.1038/s41598-020-67497-2)
Supplement: Supplementary file 3 — Supplementary file3 [file 41598_2020_67497_MOESM3_ESM.pptx]

## Slide 1
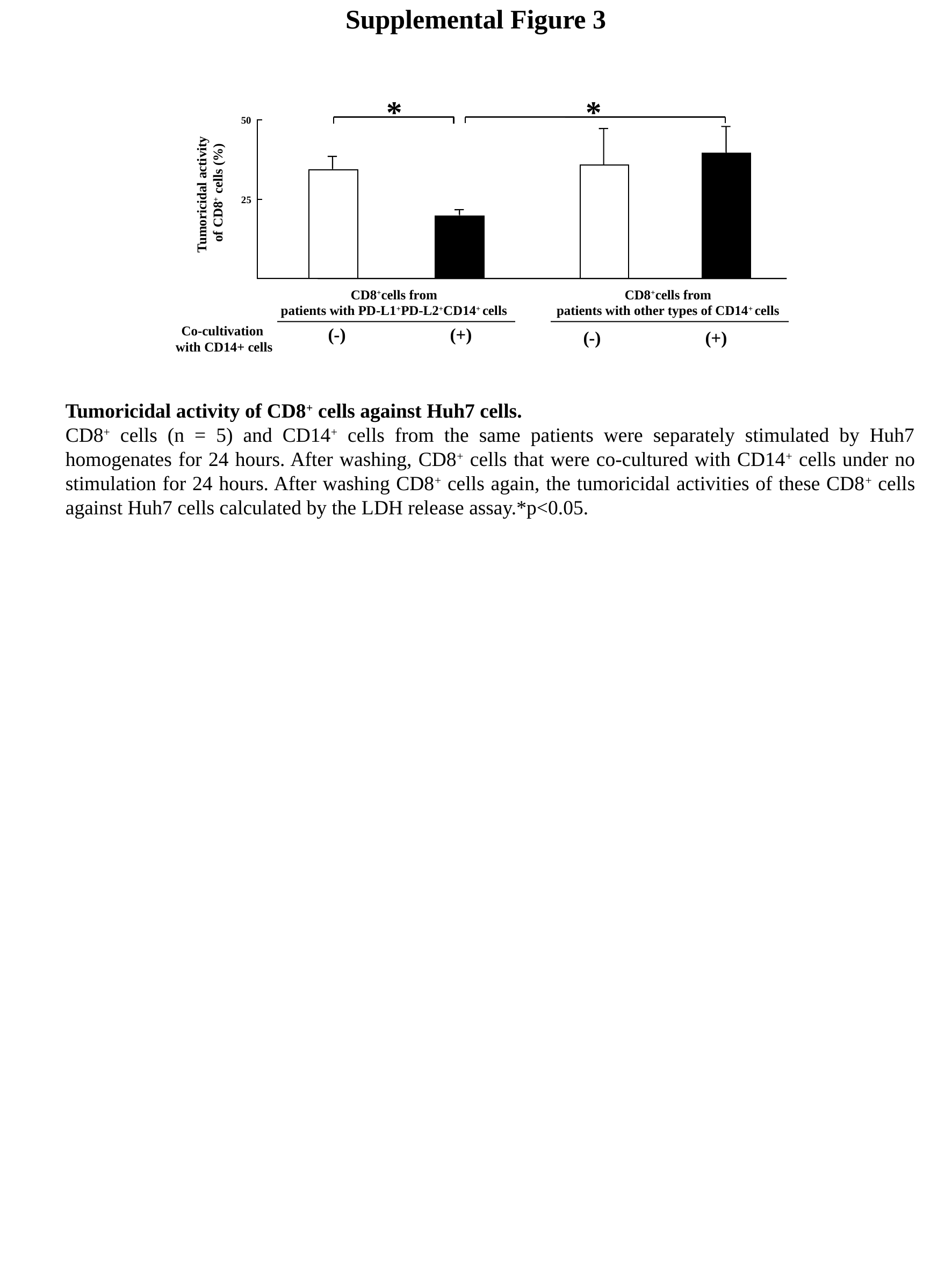

Supplemental Figure 3
*
*
50
Tumoricidal activity
 of CD8+ cells (%)
25
CD8+cells from
patients with PD-L1+PD-L2+CD14+ cells
CD8+cells from
patients with other types of CD14+ cells
Co-cultivation
with CD14+ cells
(-)
(+)
(-)
(+)
Tumoricidal activity of CD8+ cells against Huh7 cells.
CD8+ cells (n = 5) and CD14+ cells from the same patients were separately stimulated by Huh7 homogenates for 24 hours. After washing, CD8+ cells that were co-cultured with CD14+ cells under no stimulation for 24 hours. After washing CD8+ cells again, the tumoricidal activities of these CD8+ cells against Huh7 cells calculated by the LDH release assay.*p<0.05.
