# Supplementary material for: Increased both PD–L1 and PD–L2 expressions on monocytes of patients with hepatocellular carcinoma was associated with a poor prognosis
Source: Sci Rep. 2020 Jun 25;10:10377. doi: 10.1038/s41598-020-67497-2 (PMC7316832; doi:10.1038/s41598-020-67497-2)
Supplement: Supplementary file 4 — Supplementary file4 [file 41598_2020_67497_MOESM4_ESM.pptx]

## Slide 1
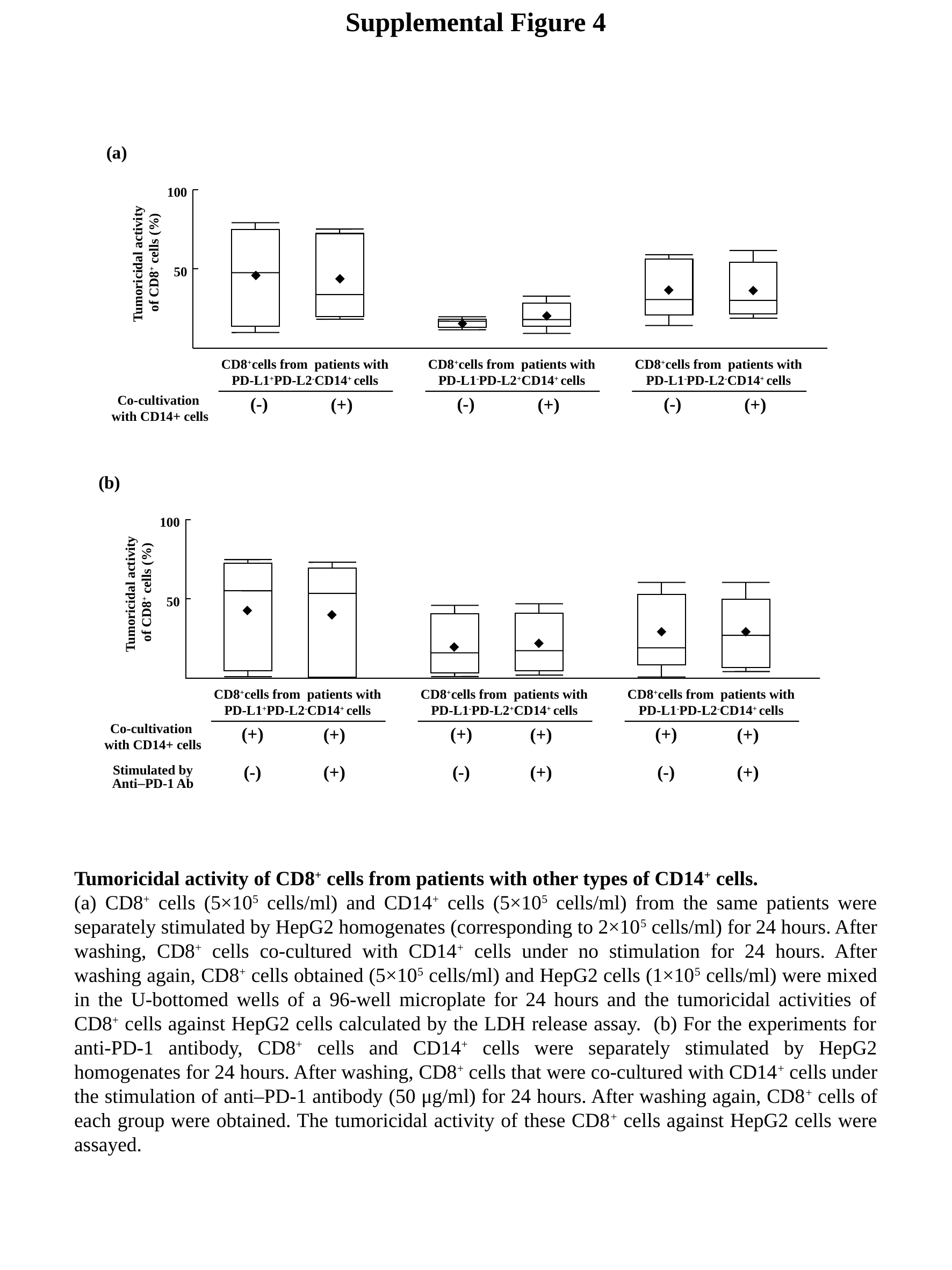

Supplemental Figure 4
(a)
100
CD8+cells from patients with
 PD-L1+PD-L2-CD14+ cells
(-)
(+)
Tumoricidal activity
 of CD8+ cells (%)
CD8+cells from patients with
 PD-L1-PD-L2-CD14+ cells
(-)
(+)
50
CD8+cells from patients with
 PD-L1-PD-L2+CD14+ cells
(-)
(+)
Co-cultivation
with CD14+ cells
(b)
100
Tumoricidal activity
 of CD8+ cells (%)
50
CD8+cells from patients with
 PD-L1+PD-L2-CD14+ cells
CD8+cells from patients with
 PD-L1-PD-L2+CD14+ cells
CD8+cells from patients with
 PD-L1-PD-L2-CD14+ cells
Co-cultivation
with CD14+ cells
(+)
(+)
(+)
(+)
(+)
(+)
Stimulated by
Anti–PD-1 Ab
(-)
(-)
(-)
(+)
(+)
(+)
Tumoricidal activity of CD8+ cells from patients with other types of CD14+ cells.
(a) CD8+ cells (5×10^5^ cells/ml) and CD14+ cells (5×10^5^ cells/ml) from the same patients were separately stimulated by HepG2 homogenates (corresponding to 2×10^5^ cells/ml) for 24 hours. After washing, CD8+ cells co-cultured with CD14+ cells under no stimulation for 24 hours. After washing again, CD8+ cells obtained (5×10^5^ cells/ml) and HepG2 cells (1×10^5^ cells/ml) were mixed in the U-bottomed wells of a 96-well microplate for 24 hours and the tumoricidal activities of CD8+ cells against HepG2 cells calculated by the LDH release assay. (b) For the experiments for anti-PD-1 antibody, CD8+ cells and CD14+ cells were separately stimulated by HepG2 homogenates for 24 hours. After washing, CD8+ cells that were co-cultured with CD14+ cells under the stimulation of anti–PD-1 antibody (50 μg/ml) for 24 hours. After washing again, CD8+ cells of each group were obtained. The tumoricidal activity of these CD8+ cells against HepG2 cells were assayed.
