# Supplementary material for: Increased both PD–L1 and PD–L2 expressions on monocytes of patients with hepatocellular carcinoma was associated with a poor prognosis
Source: Sci Rep. 2020 Jun 25;10:10377. doi: 10.1038/s41598-020-67497-2 (PMC7316832; doi:10.1038/s41598-020-67497-2)
Supplement: Supplementary file 5 — Supplementary file5 [file 41598_2020_67497_MOESM5_ESM.pptx]

## Slide 1
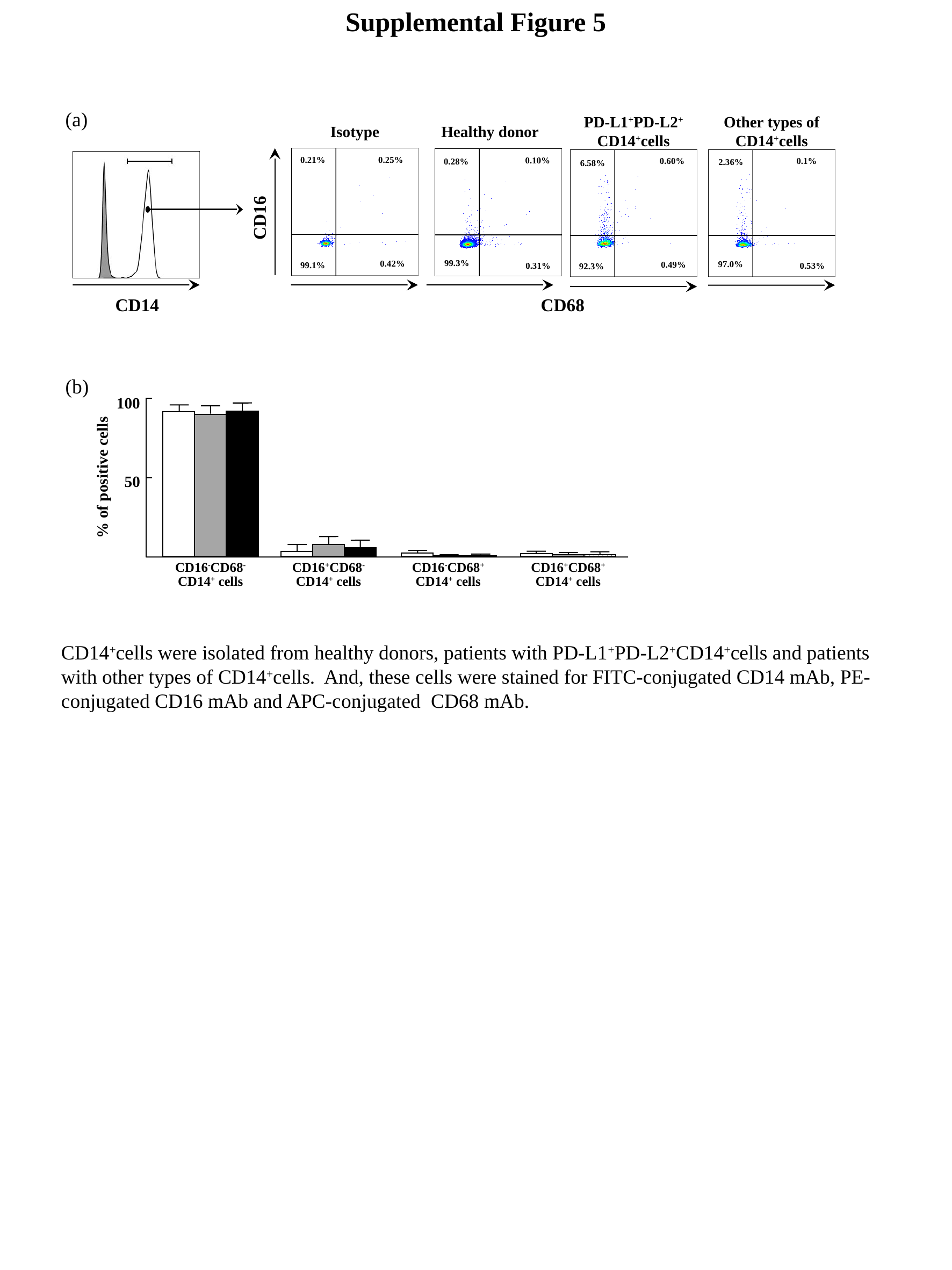

Supplemental Figure 5
(a)
PD-L1+PD-L2+
CD14+cells
Other types of CD14+cells
Isotype
Healthy donor
0.21%
0.25%
0.10%
0.60%
0.1%
0.28%
2.36%
6.58%
CD16
99.3%
0.42%
97.0%
0.49%
99.1%
0.31%
0.53%
92.3%
CD14
CD68
(b)
100
% of positive cells
50
CD16-CD68-
CD14+ cells
CD16+CD68-
CD14+ cells
CD16-CD68+
CD14+ cells
CD16+CD68+
CD14+ cells
CD14+cells were isolated from healthy donors, patients with PD-L1+PD-L2+CD14+cells and patients with other types of CD14+cells. And, these cells were stained for FITC-conjugated CD14 mAb, PE-conjugated CD16 mAb and APC-conjugated CD68 mAb.
